# Supplementary material for: Heparin-based hydrogel scaffolding alters the transcriptomic profile and increases the chemoresistance of MDA-MB-231 triple-negative breast cancer cells
Source: Biomater Sci. 2020 Feb 13;8(10):2786–96. doi: 10.1039/c9bm01481k (PMC7497406; doi:10.1039/c9bm01481k)
Supplement: Supplementary file 2 [file BM-008-C9BM01481K-s002.zip › Supplementary File 4/HP-B hydrogel + EGF (s)vControl/Pathways/my_analysis.Gsea.1545201170987/HALLMARK_APOPTOSIS.html]

Details for gene set HALLMARK\_APOPTOSIS[GSEA]

|  || Dataset | expr.class.cls#HYDEGF\_versus\_CONTROL.class.cls#HYDEGF\_versus\_CONTROL\_repos |
| Phenotype | class.cls#HYDEGF\_versus\_CONTROL\_repos |
| Upregulated in class | CONTROL |
| GeneSet | HALLMARK\_APOPTOSIS |
| Enrichment Score (ES) | -0.42570043 |
| Normalized Enrichment Score (NES) | -1.9585303 |
| Nominal p-value | 0.0 |
| FDR q-value | 3.1089154E-4 |
| FWER p-Value | 0.002 |
Table: GSEA Results Summary

  

Fig 1: Enrichment plot: HALLMARK\_APOPTOSIS      
 Profile of the Running ES Score & Positions of GeneSet Members on the Rank Ordered List

  

| PROBE | DESCRIPTION (from dataset) | GENE SYMBOL | GENE\_TITLE | RANK IN GENE LIST | RANK METRIC SCORE | RUNNING ES | CORE ENRICHMENT || 1 | ATF3 | na |  |  | 473 | 2.154 | -0.0093 | No |
| 2 | DIABLO | na |  |  | 478 | 2.150 | 0.0060 | No |
| 3 | GADD45B | na |  |  | 524 | 2.096 | 0.0187 | No |
| 4 | SMAD7 | na |  |  | 606 | 2.006 | 0.0290 | No |
| 5 | SPTAN1 | na |  |  | 1042 | 1.658 | 0.0181 | No |
| 6 | WEE1 | na |  |  | 1066 | 1.644 | 0.0287 | No |
| 7 | TNFRSF12A | na |  |  | 1130 | 1.611 | 0.0370 | No |
| 8 | HMGB2 | na |  |  | 1230 | 1.550 | 0.0430 | No |
| 9 | CREBBP | na |  |  | 1346 | 1.495 | 0.0477 | No |
| 10 | CCNA1 | na |  |  | 1442 | 1.452 | 0.0532 | No |
| 11 | LEF1 | na |  |  | 2438 | 1.143 | 0.0093 | No |
| 12 | EBP | na |  |  | 2627 | 1.097 | 0.0073 | No |
| 13 | CCND1 | na |  |  | 2684 | 1.084 | 0.0122 | No |
| 14 | PTK2 | na |  |  | 2705 | 1.079 | 0.0189 | No |
| 15 | DDIT3 | na |  |  | 2748 | 1.066 | 0.0244 | No |
| 16 | GADD45A | na |  |  | 2798 | 1.052 | 0.0294 | No |
| 17 | ERBB2 | na |  |  | 3000 | 1.004 | 0.0261 | No |
| 18 | DFFA | na |  |  | 3072 | 0.987 | 0.0295 | No |
| 19 | CTH | na |  |  | 3158 | 0.969 | 0.0320 | No |
| 20 | MADD | na |  |  | 3577 | 0.888 | 0.0165 | No |
| 21 | CDK2 | na |  |  | 3627 | 0.880 | 0.0202 | No |
| 22 | RHOT2 | na |  |  | 3705 | 0.863 | 0.0224 | No |
| 23 | BAX | na |  |  | 3746 | 0.856 | 0.0265 | No |
| 24 | ROCK1 | na |  |  | 3850 | 0.838 | 0.0271 | No |
| 25 | CASP2 | na |  |  | 4003 | 0.810 | 0.0250 | No |
| 26 | PSEN2 | na |  |  | 4592 | 0.722 | -0.0006 | No |
| 27 | PLCB2 | na |  |  | 4613 | 0.719 | 0.0035 | No |
| 28 | FDXR | na |  |  | 4868 | 0.674 | -0.0050 | No |
| 29 | DPYD | na |  |  | 5047 | 0.645 | -0.0097 | No |
| 30 | CD44 | na |  |  | 5293 | 0.607 | -0.0182 | No |
| 31 | CDC25B | na |  |  | 5390 | 0.592 | -0.0189 | No |
| 32 | JUN | na |  |  | 5642 | 0.555 | -0.0281 | No |
| 33 | SLC20A1 | na |  |  | 6250 | 0.469 | -0.0566 | No |
| 34 | BRCA1 | na |  |  | 6597 | 0.424 | -0.0717 | No |
| 35 | PPP2R5B | na |  |  | 6949 | 0.379 | -0.0873 | No |
| 36 | DAP3 | na |  |  | 7047 | 0.366 | -0.0898 | No |
| 37 | PDGFRB | na |  |  | 7303 | 0.329 | -0.1008 | No |
| 38 | TGFBR3 | na |  |  | 7326 | 0.326 | -0.0996 | No |
| 39 | IL18 | na |  |  | 7482 | 0.306 | -0.1055 | No |
| 40 | EGR3 | na |  |  | 7749 | 0.271 | -0.1175 | No |
| 41 | RARA | na |  |  | 8781 | 0.154 | -0.1705 | No |
| 42 | PEA15 | na |  |  | 9631 | 0.057 | -0.2147 | No |
| 43 | MCL1 | na |  |  | 10007 | 0.019 | -0.2342 | No |
| 44 | BCL2L2 | na |  |  | 10286 | -0.019 | -0.2486 | No |
| 45 | HMOX1 | na |  |  | 10412 | -0.037 | -0.2549 | No |
| 46 | GSTM1 | na |  |  | 10755 | -0.073 | -0.2724 | No |
| 47 | CASP8 | na |  |  | 11048 | -0.114 | -0.2869 | No |
| 48 | IRF1 | na |  |  | 11060 | -0.115 | -0.2866 | No |
| 49 | CYLD | na |  |  | 11255 | -0.131 | -0.2958 | No |
| 50 | CAV1 | na |  |  | 11391 | -0.145 | -0.3019 | No |
| 51 | KRT18 | na |  |  | 11421 | -0.149 | -0.3023 | No |
| 52 | ADD1 | na |  |  | 11543 | -0.167 | -0.3075 | No |
| 53 | PAK1 | na |  |  | 11788 | -0.201 | -0.3188 | No |
| 54 | FEZ1 | na |  |  | 11843 | -0.210 | -0.3201 | No |
| 55 | RHOB | na |  |  | 11865 | -0.214 | -0.3197 | No |
| 56 | CFLAR | na |  |  | 12192 | -0.242 | -0.3351 | No |
| 57 | ERBB3 | na |  |  | 12288 | -0.259 | -0.3382 | No |
| 58 | H1F0 | na |  |  | 12323 | -0.265 | -0.3381 | No |
| 59 | CASP1 | na |  |  | 12497 | -0.286 | -0.3451 | No |
| 60 | RETSAT | na |  |  | 12706 | -0.315 | -0.3537 | No |
| 61 | TOP2A | na |  |  | 12732 | -0.319 | -0.3527 | No |
| 62 | DNM1L | na |  |  | 13067 | -0.352 | -0.3677 | No |
| 63 | NEFH | na |  |  | 13072 | -0.352 | -0.3654 | No |
| 64 | TIMP2 | na |  |  | 13099 | -0.353 | -0.3642 | No |
| 65 | PPT1 | na |  |  | 13258 | -0.379 | -0.3698 | No |
| 66 | PMAIP1 | na |  |  | 13499 | -0.413 | -0.3794 | No |
| 67 | ANKH | na |  |  | 13596 | -0.424 | -0.3814 | No |
| 68 | FAS | na |  |  | 13787 | -0.456 | -0.3881 | No |
| 69 | SQSTM1 | na |  |  | 13980 | -0.486 | -0.3946 | No |
| 70 | GNA15 | na |  |  | 14012 | -0.490 | -0.3927 | No |
| 71 | BTG2 | na |  |  | 14115 | -0.493 | -0.3945 | No |
| 72 | RELA | na |  |  | 14142 | -0.496 | -0.3923 | No |
| 73 | BCL2L1 | na |  |  | 14288 | -0.519 | -0.3962 | No |
| 74 | GPX4 | na |  |  | 14401 | -0.538 | -0.3982 | No |
| 75 | GPX1 | na |  |  | 14583 | -0.572 | -0.4035 | No |
| 76 | BIK | na |  |  | 14637 | -0.578 | -0.4022 | No |
| 77 | CCND2 | na |  |  | 14645 | -0.580 | -0.3983 | No |
| 78 | BMF | na |  |  | 14669 | -0.585 | -0.3953 | No |
| 79 | BTG3 | na |  |  | 14693 | -0.589 | -0.3923 | No |
| 80 | ENO2 | na |  |  | 14703 | -0.591 | -0.3885 | No |
| 81 | GCH1 | na |  |  | 14797 | -0.603 | -0.3891 | No |
| 82 | ETF1 | na |  |  | 14811 | -0.605 | -0.3854 | No |
| 83 | TNF | na |  |  | 14846 | -0.611 | -0.3827 | No |
| 84 | CDKN1A | na |  |  | 14882 | -0.616 | -0.3801 | No |
| 85 | BCAP31 | na |  |  | 15001 | -0.633 | -0.3818 | No |
| 86 | CASP7 | na |  |  | 15674 | -0.757 | -0.4116 | No |
| 87 | CDKN1B | na |  |  | 15677 | -0.758 | -0.4062 | No |
| 88 | ANXA1 | na |  |  | 15923 | -0.806 | -0.4133 | No |
| 89 | DAP | na |  |  | 16126 | -0.851 | -0.4177 | No |
| 90 | CASP9 | na |  |  | 16279 | -0.893 | -0.4193 | Yes |
| 91 | TSPO | na |  |  | 16294 | -0.896 | -0.4135 | Yes |
| 92 | GSN | na |  |  | 16299 | -0.897 | -0.4073 | Yes |
| 93 | IL1A | na |  |  | 16315 | -0.902 | -0.4016 | Yes |
| 94 | BCL2L11 | na |  |  | 16361 | -0.913 | -0.3973 | Yes |
| 95 | CD14 | na |  |  | 16500 | -0.948 | -0.3978 | Yes |
| 96 | GSR | na |  |  | 16589 | -0.967 | -0.3954 | Yes |
| 97 | IGF2R | na |  |  | 16595 | -0.968 | -0.3887 | Yes |
| 98 | TGFB2 | na |  |  | 16607 | -0.972 | -0.3823 | Yes |
| 99 | TIMP1 | na |  |  | 16662 | -0.990 | -0.3780 | Yes |
| 100 | GPX3 | na |  |  | 16764 | -1.015 | -0.3759 | Yes |
| 101 | F2R | na |  |  | 16819 | -1.032 | -0.3713 | Yes |
| 102 | VDAC2 | na |  |  | 16846 | -1.042 | -0.3652 | Yes |
| 103 | PLAT | na |  |  | 16888 | -1.050 | -0.3598 | Yes |
| 104 | IFITM3 | na |  |  | 16890 | -1.051 | -0.3523 | Yes |
| 105 | TAP1 | na |  |  | 16995 | -1.089 | -0.3499 | Yes |
| 106 | BID | na |  |  | 17292 | -1.178 | -0.3569 | Yes |
| 107 | EMP1 | na |  |  | 17346 | -1.196 | -0.3511 | Yes |
| 108 | CASP3 | na |  |  | 17375 | -1.207 | -0.3438 | Yes |
| 109 | IGFBP6 | na |  |  | 17518 | -1.266 | -0.3422 | Yes |
| 110 | HSPB1 | na |  |  | 17580 | -1.287 | -0.3361 | Yes |
| 111 | PSEN1 | na |  |  | 17614 | -1.300 | -0.3284 | Yes |
| 112 | TNFSF10 | na |  |  | 17691 | -1.328 | -0.3229 | Yes |
| 113 | CASP4 | na |  |  | 17716 | -1.342 | -0.3144 | Yes |
| 114 | NEDD9 | na |  |  | 17722 | -1.343 | -0.3050 | Yes |
| 115 | PPP3R1 | na |  |  | 17792 | -1.378 | -0.2987 | Yes |
| 116 | ISG20 | na |  |  | 17840 | -1.410 | -0.2910 | Yes |
| 117 | BCL10 | na |  |  | 17922 | -1.452 | -0.2848 | Yes |
| 118 | CTNNB1 | na |  |  | 17952 | -1.465 | -0.2758 | Yes |
| 119 | IL1B | na |  |  | 18115 | -1.556 | -0.2730 | Yes |
| 120 | IL6 | na |  |  | 18227 | -1.619 | -0.2672 | Yes |
| 121 | XIAP | na |  |  | 18281 | -1.662 | -0.2580 | Yes |
| 122 | RNASEL | na |  |  | 18292 | -1.673 | -0.2465 | Yes |
| 123 | LGALS3 | na |  |  | 18514 | -1.848 | -0.2447 | Yes |
| 124 | PDCD4 | na |  |  | 18548 | -1.865 | -0.2330 | Yes |
| 125 | CLU | na |  |  | 18586 | -1.909 | -0.2212 | Yes |
| 126 | BMP2 | na |  |  | 18629 | -1.962 | -0.2093 | Yes |
| 127 | DNAJA1 | na |  |  | 18633 | -1.967 | -0.1953 | Yes |
| 128 | EREG | na |  |  | 18655 | -1.994 | -0.1820 | Yes |
| 129 | APP | na |  |  | 18704 | -2.052 | -0.1697 | Yes |
| 130 | TIMP3 | na |  |  | 18706 | -2.053 | -0.1550 | Yes |
| 131 | DNAJC3 | na |  |  | 18716 | -2.072 | -0.1405 | Yes |
| 132 | SOD1 | na |  |  | 18910 | -2.370 | -0.1335 | Yes |
| 133 | IFNGR1 | na |  |  | 18912 | -2.376 | -0.1165 | Yes |
| 134 | BIRC3 | na |  |  | 18942 | -2.435 | -0.1004 | Yes |
| 135 | CASP6 | na |  |  | 18952 | -2.470 | -0.0831 | Yes |
| 136 | BNIP3L | na |  |  | 18957 | -2.475 | -0.0655 | Yes |
| 137 | TXNIP | na |  |  | 18972 | -2.510 | -0.0481 | Yes |
| 138 | SOD2 | na |  |  | 18995 | -2.569 | -0.0308 | Yes |
| 139 | SATB1 | na |  |  | 19008 | -2.600 | -0.0126 | Yes |
| 140 | SAT1 | na |  |  | 19134 | -3.131 | 0.0034 | Yes |
Table: GSEA details [plain text format]

  

Fig 2: HALLMARK\_APOPTOSIS      
 Blue-Pink O' Gram in the Space of the Analyzed GeneSet

  

Fig 3: HALLMARK\_APOPTOSIS: Random ES distribution      
 Gene set null distribution of ES for **HALLMARK\_APOPTOSIS**
